# Food insecurity was negatively associated with adherence to the “fruits, vegetables, and foods rich in animal protein” dietary pattern among university students’ households: the 2018 Mexican National Household Survey

**DOI:** 10.1186/s12889-023-15755-z

**Published:** 2023-05-11

**Authors:** Alejandra Betancourt-Núñez, Pablo Alejandro Nava-Amante, María Fernanda Bernal-Orozco, Barbara Vizmanos, Elisa J. Vargas-García, Fabiola Márquez-Sandoval, Miguel Amaury Salas-García, Andrés Díaz-López

**Affiliations:** 1 Doctorado en Ciencias de la Nutrición Traslacional, Centro Universitario de Ciencias de la Salud (CUCS), Universidad de Guadalajara (UdeG), Guadalajara, México; 2 Laboratorio de Evaluación del Estado Nutricio, CUCS, UdeG, Guadalajara, México; 3 Instituto Traslacional de Nutrigenética y Nutrigenómica, CUCS, UdeG, Guadalajara, México; 4 Departamento de Disciplinas Filosófico, Metodológicas e Instrumentales, CUCS, UdeG, Guadalajara, Jalisco México; 5 Human Nutrition, School of Medicine, Dentistry and Nursing, College of Medical, Veterinary, and Life Sciences, University of Glasgow, Glasgow, UK; 6 Serra Hunter Fellow, Universitat Rovira I Virgili (URV), Reus, Spain; 7 Nutrition and Mental Health Research Group (NUTRISAM), Rovira I Virgili University (URV), Reus, Spain; 8 Institut d’Investigació Sanitària Pere Virgili (IISPV), Reus, Spain; 9 Centro de Investigación Biomédica en Red Fisiopatología de la Obesidad y la Nutrición (CIBEROBN), Institute of Health Carlos III, Madrid, Spain

**Keywords:** Dietary pattern, Food insecurity, Food security, College student, University student

## Abstract

**Background:**

University students are often affected by food insecurity (FI) and this situation has been associated with low consumption of fruit/vegetables and high intake of added sugars and sweet drinks. However, there needs to be more evidence on the association between FI and dietary patterns (DPs), assessing the overall diet and allowing analysis of commonly consumed food combinations. We aimed to analyze the association between FI and DPs in university students’ households.

**Methods:**

We used data from 7659 university student households from the 2018 Mexican National Household Income and Expenditure Survey (ENIGH, for its acronym in Spanish). We obtained FI levels (mild, moderate, and severe) using the validated Mexican Food Security Scale (EMSA, Spanish acronym). Two DPs were identified by principal component analysis based on the weekly frequency of consumption of 12 food groups. Multivariate logistic regression adjusted by university student and household’s characteristics was applied.

**Results:**

Compared to food security, households with mild-FI (OR:0.34; 95%CI:0.30, 0.40), moderate-FI (OR:0.20; 95%CI:0.16, 0.24) or severe-FI (OR:0.14; 95%CI:0.11, 0.19) were less likely to adhere to the dietary pattern “Fruits, vegetables and foods rich in animal protein” (fruits, vegetables, meat, fish or seafood, dairy products, and starchy vegetables). In addition, people with severe-FI (OR:0.51; 95% CI:0.34, 0.76) were also less likely to adhere to the dietary pattern “Traditional-Westernized” (pulses, oils or fats, sugar, sweets, industrialized drinks, foods made from corn/maize, wheat, rice, oats or bran, coffee, tea and eggs).

**Conclusions:**

In these households FI impairs the ability to consume a healthy dietary pattern (fruits/vegetables and foods rich in animal protein). In addition, the intake of foods typical of the Mexican food culture reflecting the local Western dietary pattern is compromised in households with severe-FI.

**Supplementary Information:**

The online version contains supplementary material available at 10.1186/s12889-023-15755-z.

## Background

According to the Food and Agriculture Organization of the United Nations (FAO), food insecurity (FI) is “the lack of regular access to enough safe and nutritious food for normal or average growth and development and active, healthy life. It may be due to unavailability of food or lack of resources to obtain food” [1]. In 2018, the global prevalence of moderate and severe FI was 25.9%, which increased to 30.4% in 2020 because of the coronavirus pandemic (COVID-19) [2]. In particular, university students are among the groups most affected by FI. A review published in 2020 showed that the overall FI prevalence in college students from the United States of America (USA) was 41% (ranging from 10 to 75%) [3]. Similarly, in another review published in 2017, the overall FI prevalence in university students from the USA, South Africa, Australia, Canada, and Malaysia was 42% (12.5% to 84%) [4]. Furthermore, in 2020, the prevalence of FI in university students changed after COVID-19 [5, 6], and in some cases, this prevalence increased [5].

Some characteristics of college students that have been associated with FI are: belonging to a race/ethnic minority [4, 7, 8]; having financial, food, and housing independence from parents [4]; having children [4]; being employed (part/full time) [8–10]; living off-campus [9, 10]; having a low socioeconomic status [8], and having a female head of household [8]. Also, students with FI were more likely to rent a house [11] and to have parents whose highest level of education was high school or less [8, 11].

In university students, FI has been associated with lower academic performance [4, 11–13], unhealthy lifestyles [11, 12, 14, 15], and health problems [4, 9–11, 13, 14, 16]. In this regard, FI has been associated with lower grade point average (GPA) [4, 11–13], increased difficulty concentrating in class, greater likelihood of dropping out of institutions [4], fewer days of physical activity [14], fewer days of sufficient sleep [11, 14] or poor sleep quality [12], being classified as highly stressed [11, 12], lower self-reported general health [4, 10, 13, 14, 16], higher prevalence of disordered eating behaviors [12, 15], and obesity (BMI > 30) [9, 11, 13, 14]. In addition, students with FI are more likely to: use tobacco or marijuana and binge drink, have a diagnosis of depression, and experience more stressful life events (i.e., conflicts with roommate/housemates and/or parents, excessive debts, lack of health coverage, among others) [11].

Regarding food intake, FI has been associated with consuming an unhealthy diet within this vulnerable population. College students with FI have a lower intake of fruits and vegetables [4, 9, 14, 17], whole grains, dairy products, dietary calcium, meat or other protein rich foods [17], and a lower consumption of dinner and breakfast [17]. On the other hand, they have a higher intake of sugar-sweetened drinks [7, 9, 11, 17], total added sugars [7, 9, 17] and fast food [11, 17]. Furthermore, university students with severe FI have shown significantly lower adherence to the Mediterranean diet assessed as a food index [17, 18].

However, to our knowledge, there are no studies on the association between FI and dietary patterns (DPs) as generated by multivariate analysis among college students, although it has been analyzed in other population groups [19–23]. This approach is becoming increasingly popular for assessing dietary habits strongly associated with a population’s culture, and diet contents in a specific pattern. The assessment of DPs allows the analysis of the totality of the diet and the combinations of foods consumed regularly, considering that people/individuals do not usually consume one food or nutrient in isolation [24]. On the other hand, college students are considered emerging adults; that is, they are transitioning to adulthood. They are considered emerging adults because they generally do not have the normative responsibilities of adulthood (e.g., at this stage, people decide whether or not to work, whether or not to live with their parents, whether or not to be financially independent, etc.). At this stage of life, they often have a combination of adult dependence and independence (period of semi-autonomy) because they assume some responsibilities of independent living but leave others to parents, university authorities, or other adults. In particular, this is considered a period of change and exploration, and their living situations are diverse [25, 26]. In addition, transitioning into university life favors a lifestyle characterized by high levels of stress, poor sleep, physical inactivity, and changes in daily eating towards an unhealthy Western-type diet [17, 27–29]. These results will allow us to identify to what extent the FI situation affects the DPs of households with university students, in which it would be necessary to find ways to improve the dietary pattern (PD) to make it healthier, if not healthier. Therefore, this study aimed to analyze the association between FI and DPs in university students’ households for the first time to our knowledge.

## Methods

### Study design and data source

Open access data from the 2018 Mexican National Household Income and Expenditure Survey (Encuesta Nacional de Ingresos y Gastos de los Hogares; *ENIGH*) was analyzed [30]. The detailed methodology of *ENIGH* 2018 has been described previously [31]. In brief, the *ENIGH* 2018 is a cross-sectional survey carried out by The National Institute of Statistics and Geography (abbreviated *INEGI* in Spanish) in collaboration with the National Council for the Evaluation of the Social Development Policy (abbreviated *CONEVAL* in Spanish). This survey provides information on the occupational and sociodemographic characteristics of household members. It identifies households that have problems meeting their food needs due to a lack of money or other resources (production for self-consumption, food barter, food aid programs, and/or donations, among others).

The sampling design was probabilistic, two-stage stratified cluster sampling. The dwelling was the sampling unit, the household was the observation unit, and the residence, the family, and its members were the unit of analysis. Therefore, this survey represents households throughout the country (urban and rural). Trained staff visited homes to administer the standardized questionnaires through face-to-face interviews. Specifically, the household and dwelling questionnaire, whose variables are analyzed in this paper, was either answered by the head of the household, the partner, or a household member aged 18 or older who could provide the information requested in the questionnaire. The *ENIGH* 2018 fieldwork occurred from 21 August to 28 November [31].

For this analysis, we drew on open-access information on food access for 74,647 households and data on 8991 university students, from which we selected households with at least one university student present at the time of *ENIGH* 2018 data collection, leaving a total of 7663 families. Some households had more than one university student living in them; however, we only analyzed data from one university student per household. Two databases (with participants´ and household characteristics) were linked and matched the data of each university student (whoever is) to their corresponding household. Therefore, student selection was unintentional. Finally, we eliminated four homes because they had no food consumption data (*n* = 7659).

*ENIGH* 2018 did not publish any identifiable data. The *INEGI* makes the data available to society, safeguarding the principles of confidentiality. The INEGI works under the Law of the National System of Statistical Information and Geography in force, which in Article 37 indicates that the data provided for statistical purposes are strictly confidential. Therefore, the present study did not require Ethics Committee approval. Informed consent was obtained from all individual participants included in the study by the *ENIGH* team [32].

### Sociodemographic characteristics 

We analyzed the following university students’ characteristics: age, sex, marital status [partnered (living together, cohabiting or married); not in a relationship (single, separated, divorced, or widowed)], type of university in which the student was enrolled (private or public), student’s school year (first to the fifth school year), whether the student received a scholarship in the current scholar year (yes or no), indigenous language (yes or no); whether the students considered themselves as indigenous according to their culture (yes or no), and whether they had worked in the month before completing the survey (yes or no).

The following household characteristics were selected for analysis: 1) age, 2) sex, and 3) level of education of the household head (bachelor's degree or above; elementary to high school; incomplete elementary school or less); 4) presence of children under 18 in the household (yes or no); 5) household types [nuclear (one primary family group), extended (the head of household and the primary family group plus other family groups or relatives), and others (single person household -household consisting of only one person who is the head of household-; composite household -nuclear or extended household plus persons unrelated to the household’s head-; and co-residential household -two or more persons unrelated to the head of household-)], 6) socioeconomic status (high, upper-middle, lower-middle, or low), and 7) type of locality according to the number of residents where the household is located (metropolitan area: ≥ 100,000, urban area: 2,500 to 99,999, and rural area: < 2,500). *INEGI* classified socioeconomic status by analyzing household characteristics, amenities and furnishings [33].

### Household’s food security status 

We selected from the database the questions constituting the validated Mexican Food Security Scale (*EMSA* in Spanish). The *EMSA* collects people’s situations/experiences about difficulty accessing food due to a lack of money or other resources in the last three months. The *EMSA* includes 12 questions with yes/no response options. The first six questions aim to determine the food access experience of the adults living in the household. The remaining six questions ask about the food access experience of children under 18 living in the home. If no children under 18 live in the household, only the first six questions on the scale are answered. One point is assigned for each affirmative response. The questionnaire is interpreted as follows for adult-only households and households with children under 18: household food security (zero affirmative answers), household with mild-FI (1–2 or 1–3 affirmative answers, respectively), household with moderate-FI (3–4 or 4–7 affirmative responses, respectively), household with severe-FI (5–6 or 8–12 affirmative answers, respectively) [34].

### Food intake

We analyzed the weekly frequency of consumption of 12 food groups (Table 1) prepared and consumed at home. To obtain these data, the interviewer began with the following sentence: “Now, I would like to ask about the types of foods that you or any of the household members ate during the last seven days.” Then the interviewer asked: “During the last seven days, how many days did you eat… (each of the 12 food groups)?” Response options ranged from 0 to 7 days. We categorized response options as follows: 0 days per week, 1 to 3 days per week, 4 to 6 days per week, and seven days per week (daily).

**Table 1:** Foods considered in each food group

Food group	Foods
Foods made from corn, wheat, rice, oats or bran	Tortilla, corn dough food (*masa*), bread (white bread, whole wheat bread, tin loaf, rustic bread, sweet bread), cookies, breakfast cereals, pasta for soup or any other food made from corn, wheat, rice, oats or bran
Roots or starchy vegetables	Potatoes, sweet potatoes, or any other food that come from roots or starchy vegetables
Vegetables	Fresh, canned, or dried vegetables, or vegetables in stews, soups, or sauces
Fruits	Fresh, canned, or dried fruits, or fruits in desserts, or salads
Meat	Beef, pork, lamb, goat, rabbit, poultry, or liver, kidney, heart, and other viscera
Eggs	Chicken, quail, duck eggs or other bird eggs
Fish or seafood	Fresh or dried fish, sardines, tuna, shrimp or any other seafood
Legumes or seeds	Foods based on beans, lentils, broad beans, chickpeas, soybeans, peanuts, *pepitas*, granola, *palanquetas*, walnuts, amaranth, or nuts
Dairy products	Cheese, yogurt, milk, or any other dairy products
Oils or fats	Any type of oil (soy, safflower, canola, sunflower, corn), butter, peanut butter, mayonnaise, cream, or lard
Sugar, sweets, soft drinks or industrialized beverages	Sugar or honey (from bee or maple), corn syrup, jelly, jam, *cajeta* (traditional sweet), powder to prepare drinks, *flan*, candies, chocolates, soft drinks, or industrialized beverages
Condiments, coffee or tea	Condiments, coffee, tea

The name of the food, as it is known in Mexico, is shown in italics

Participants also indicated if consumption in the last week was the same, higher, or lower than usual.

### Statistical analysis

Categorical variables are presented as frequency and percentage, while continuous variables as mean and standard deviation. Chi-square, Fisher’s exact, independent Student t-test and one-factor ANOVA were used, as appropriate, to test differences in characteristics across categories.

The DPs were identified by the multivariate statistical test of Principal Components Analysis (PCA) using the weekly frequency of consumption of 12 food groups (response options 0 to 7). The Bartlett test of sphericity (statistical significance *p* < 0.05) and the Kaiser–Meyer–Olkin test (score = 0.701) were conducted a priori to verify the feasibility of this analysis [35]. We selected the number of factors/DPs among the study population based on the Scree plot and considering the number of foods in the DPs; each was expected to have no less than three items [36]. We used a Varimax orthogonal rotation to improve the interpretability of the DPs. Food groups with a factor loading ≥ 0.3 (minimum significance) were considered the significant foods associated with the DPs [36]. Each DP was named according to the foods it consisted of.

Next, the adherence percentage to each DP was calculated as follows: responses to each food group ranged from 0 to 7 (days of consumption per week), and each DP considered up to 6 food groups (as will be detailed in the Results section). Therefore, each participant could obtain a maximum score of 42 in each DP (6 food groups*7 days per week = 42) equivalent to 100% adherence to the DP. The adherence percentage to each DP was calculated as follows: [(sum of the frequency of consumption of the six foods groups that make up a DP * 100) / 42].

Additionally, we performed a hierarchical cluster analysis based on the variables related to the percentage of adherence to each DP. Ward's method and block distance were applied. Subsequently, we used discriminant analysis to assess the stability of the cluster solution.

A scatter plot of the percentage of adherence to each DP was prepared differentiating between participants with and without FI, and differentiating the membership of each cluster.

The adherence percentage to each DP was also divided into two categories for the association analyses: ≤ 50% was classified as non-adherence and ≥ 51% was classified as adherence to each DP. For the cluster analyses, it was also assessed into three categories classifying ≤ 50% as non-adherence, 51% to 75% as medium adherence, and 76% to 100% as high adherence.

To investigate the associations between university students’ household food security status (food security (reference category), mild-FI, moderate-FI and severe-FI) as the independent variable and adherence to the two identified DPs as dichotomous outcomes, we used unadjusted and multivariate adjusted logistic regression models, based on existing literature, using two sets of confounders. Model 1 was adjusted for students’ age, sex, marital status, enrolment college type, academic year, and indigenous self-identification. Model 2 was adjusted by model 1 variables plus age, sex and level of education of the head of household, household type, socioeconomic status, and type of locality. The odds ratios (ORs) and 95% confidence intervals (CIs) were calculated.

Statistical analysis was performed with STATA version 15.0 software (StataCorp, College Station, TX, USA), and a *p* value < 0.05 was considered significant. The figures were done in RStudio® and Excel.

## Results

### Participants sociodemographic characteristics 

The mean age of the university students (*n* = 7659) was 22.2 ± 5.3, and 51% were female. The minority were cohabiting (10.2%), enrolled in a private university (28.1%), had received a scholarship the last year (16%), spoke an indigenous language (1.7%), and self-identified as indigenous (22.7%). The majority were of nuclear (66.9%) or extended (28.9%) household type, had a middle socioeconomic level (77.1%), had a male head of household (70%), and the head of household had elementary to high school education (71.7%). In terms of households with FI, the overall prevalence was 30.8% (Table 2).

**Table 2:** Sociodemographic characteristics of university students

*University students’ characteristics*	*n* = 7659
Age (years), *mean (SD)*	22.2 (5.3)
Sex, *n (%)*	
Man	3756 (49.0)
Woman	3903 (51.0)
Marital status	
Partnered (living together, cohabiting or married)	782 (10.2)
Not in a relationship (single, separated, divorced, or widower)	6877 (89.8)
Enrollment college type	
Private	2156 (28.1)
Public	5503 (71.9)
Academic year	
3rd to 5th year	3211 (41.9)
2nd year	1869 (24.4)
1st year	2579 (33.7)
Scholarship student	
No	6430 (84.0)
Yes	1229 (16.0)
Employment status in the month before the survey	
No employed	4478 (58.5)
Employed	3171 (41.5)
Indigenous language	
No	7530 (98.3)
Yes	129 (1.7)
Indigenous self-identification	
No	5920 (77.3)
Yes	1739 (22.7)
*University students’ household characteristics*	
Age of the household head (years), *mean (SD)*	48.7 (12.2)
Sex of the household head, *n (%)*	
Man	5359 (70.0)
Woman	2300 (30.0)
Education of the household head	
Bachelor´s degree or more	1487 (19.4)
Elementary to high school	5492 (71.7)
Incomplete elementary school or less	680 (8.9)
Household type	
Nuclear	5124 (66.9)
Extended	2211 (28.9)
Other ^a^	324 (4.2)
Children under 18-years-old	
Yes	1967 (25.7)
No	5692 (74.3)
Type of locality	
Metropolitan (≥ 100,000 inhabitants)	4053 (52.9)
Urban (≥ 2,500 inhabitants)	1999 (26.1)
Rural (< 2,500 inhabitants)	1607 (21.0)
Socioeconomic status	
High	1032 (13.5)
Upper-middle	2048 (26.7)
Lower-middle	3859 (50.4)
Low	720 (9.4)
Household food security status ^b^	
Food security	5301 (69.2)
Mild food insecurity	1240 (16.2)
Moderate food insecurity	680 (8.9)
Severe food insecurity	438 (5.7)

Data are presented as mean (standard deviation, SD) for continuous variables and frequency (percentage) for categorical variables

^a^ Other: single-person, composite and co-residential household

^b^ According to the Mexican Food Security Scale

### Food frequency consumption according to household’s food security/insecurity status

Significant differences were observed in the consumption of all food groups according to the degree of FI, except for egg consumption. Compared to households with FI, households with food security had a higher frequency of consumption (> 4 days per week) of vegetables, fruits, meat, and dairy products. In addition, fish or seafood consumption was more frequent (at least one day per week) among the food-secure. Regardless of the degree of household food security, less than half of the households consumed fruits and vegetables daily, and more than half consumed sugar, sweets, soft drinks or industrialized beverages daily. Most participants (93%) mentioned that the frequency of food consumption in the last seven days was similar to regular consumption (Table 3).

**Table 3:** Description of weekly frequency consumption of 12 food groups, according to household’s food security/insecurity status^a^

	Household food security status				
Food groups consumption (days per week)^b^	Food security (*n* = 5301)	Mild-Food Insecurity (*n* = 1240)	Moderate-Food Insecurity (*n* = 680)	Severe-Food Insecurity (*n* = 438)	p^c^
Foods made from corn, wheat, rice, oats or bran					
0 days	11 (0.2)	4 (0.3)	1 (0.1)	3 (0.7)	< 0.001
1–3 days	201 (3.8)	37 (3.0)	45 (6.6)	38 (8.7)	
4–6 days	302 (5.7)	56 (4.5)	37 (5.4)	26 (5.9)	
Daily	4787 (90.3)	1143 (92.2)	597 (87.8)	371 (84.7)	
Roots and starchy vegetables					
0 days	526 (9.9)	183 (14.8)	151 (22.2)	117 (26.7)	< 0.001
1–3 days	3762 (71.0)	901 (72.7)	465 (68.4)	267 (61.0)	
4–6 days	518 (9.8)	82 (6.6)	34 (5.0)	29 (6.6)	
Daily	495 (9.3)	74 (6.0)	30 (4.4)	25 (5.7)	
Vegetables					
0 days	77 (1.5)	44 (3.5)	40 (5.9)	51 (11.6)	< 0.001
1–3 days	1715 (32.4)	556 (44.8)	363 (53.4)	225 (51.4)	
4–6 days	1077 (20.3)	224 (18.1)	85 (12.5)	54 (12.3)	
Daily	2432 (45.9)	416 (33.5)	192 (28.2)	108 (24.7)	
Fruits					
0 days	164 (3.1)	127 (10.2)	104 (15.3)	114 (26.0)	< 0.001
1–3 days	1585 (29.9)	600 (48.4)	385 (56.6)	238 (54.3)	
4–6 days	1042 (19.7)	201 (16.2)	74 (10.9)	27 (6.2)	
Daily	2510 (47.3)	312 (25.2)	117 (17.2)	59 (13.5)	
Meat					
0 days	73 (1.4)	59 (4.8)	45 (6.6)	70 (16.0)	< 0.001
1–3 days	2705 (51.0)	881 (71.0)	492 (72.4)	318 (72.6)	
4–6 days	1431 (27.0)	222 (17.9)	101 (14.9)	37 (8.4)	
Daily	1092 (20.6)	78 (6.3)	42 (6.2)	13 (3.0)	
Eggs					
0 days	178 (3.4)	44 (3.5)	26 (3.8)	17 (3.9)	0.119
1–3 days	2119 (40.0)	558 (45.0)	273 (40.1)	185 (42.2)	
4–6 days	1110 (20.9)	237 (19.1)	140 (20.6)	77 (17.6)	
Daily	1894 (35.7)	401 (32.3)	241 (35.4)	159 (36.3)	
Fish or seafood					
0 days	1825 (34.4)	671 (54.1)	404 (59.6)	279 (63.7)	< 0.001
1–3 days	3180 (60.0)	532 (42.9)	259 (38.1)	149 (34.0)	
4–6 days	180 (3.4)	19 (1.5)	9 (1.3)	5 (1.1)	
Daily	116 (2.2)	18 (1.5)	7 (1.0)	5 (1.1)	
Pulses or seeds					
0 days	154 (2.9)	39 (3.1)	27 (4.0)	22 (5.0)	0.011
1–3 days	1536 (29.0)	362 (29.2)	187 (27.5)	118 (26.9)	
4–6 days	872 (16.4)	162 (13.1)	116 (17.1)	56 (12.8)	
Daily	2739 (51.7)	677 (54.6)	350 (51.5)	242 (55.3)	
Dairy products					
0 days	152 (2.9)	80 (6.5)	83 (12.2)	76 (17.4)	< 0.001
1–3 days	1176 (22.2)	407 (32.8)	266 (39.1)	182 (41.6)	
4–6 days	802 (15.1)	198 (16.0)	96 (14.1)	52 (11.9)	
Daily	3171 (59.8)	555 (44.8)	235 (34.6)	128 (29.2)	
Oils or fats					
0 days	38 (0.7)	12 (1.0)	5 (0.7)	7 (1.6)	0.005
1–3 days	229 (4.3)	70 (5.6)	47 (6.9)	33 (7.5)	
4–6 days	352 (6.6)	72 (5.8)	39 (5.7)	27 (6.2)	
Daily	4682 (88.3)	1086 (87.6)	589 (86.6)	371 (84.7)	
Sugar, sweets, soft drinks or industrialized beverages					
0 days	157 (3.0)	41 (3.3)	28 (4.1)	41 (9.4)	< 0.001
1–3 days	781 (14.7)	189 (15.2)	118 (17.4)	83 (18.9)	
4–6 days	566 (10.7)	110 (8.9)	64 (9.4)	42 (9.6)	
Daily	3797 (71.6)	900 (72.6)	470 (69.1)	272 (62.1)	
Condiments, coffee or tea					
0 days	370 (7.0)	96 (7.7)	76 (11.2)	62 (14.2)	< 0.001
1–3 days	828 (15.6)	200 (16.1)	114 (16.8)	87 (19.9)	
4–6 days	476 (9.0)	114 (9.2)	70 (10.3)	38 (8.7)	
Daily	3627 (68.4)	830 (66.9)	420 (61.8)	251 (57.3)	
Reported consumption vs usual consumption is:					< 0.001
Same	5099 (96.2)	1116 (90.0)	560 (82.4)	347 (79.2)	
Higher	93 (1.8)	24 (1.9)	27 (4.0)	16 (3.7)	
Less	109 (2.1)	100 (8.1)	93 (13.7)	75 (17.1)	

Data are presented as frequency and percentage, n (%)

^a^ According to the Mexican Food Security Scale

^b^ The list of foods included in each food group can be consulted in Table 1

^c^ Association between categories was assessed with Chi-square; p < 0.05 was considered as significant

### Dietary patterns description

**Fig. 1:**
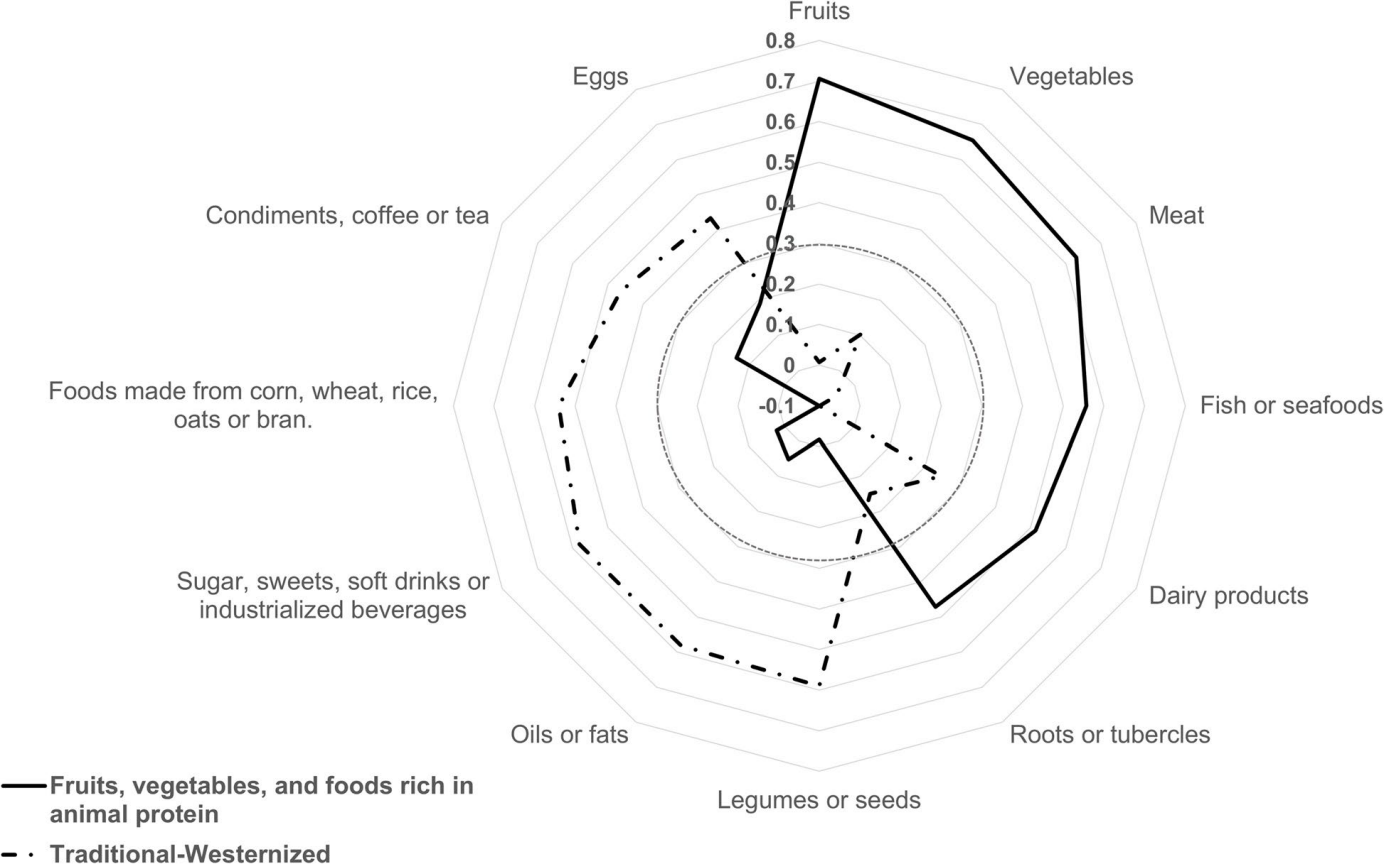
Spider plot showing the factorial loadings for each food groups for the two factors/dietary patterns. The foods included in the DP “Fruits, vegetables, and food rich in animal protein” are shown on the solid line. The foods included in the DP “Traditional-Westernized” are shown on the thick dotted line. DPs only included food groups with a factor loading ≥ 0.3. The thin dotted line indicates the factorial loading 0.3

Factor analysis revealed two DPs among study participants that explained 33.45% of the total variance. The first DP (19.70% of the total variance) was labeled “Fruits, vegetables and foods rich in animal protein” (DP FV-AP) because it consisted of fruits, vegetables, meat, fish, seafood, dairy products, roots, and starchy vegetables. The second DP (13.75% of the total variance) was labeled “Traditional-Westernized” (DP T-W) because it consisted of foods typical of the Mexican food culture, such as pulses (e.g., beans), seeds, oils, fats, dishes/foods made from corn, wheat, rice, oats or bran, eggs, condiments, coffee, and tea, as well as foods typical of Western cultures such as sugar, sweets, soft drinks, and industrialized beverages (see Fig. 1 and Additional file 1).

### Adherence to dietary patterns description

**Fig. 2:**
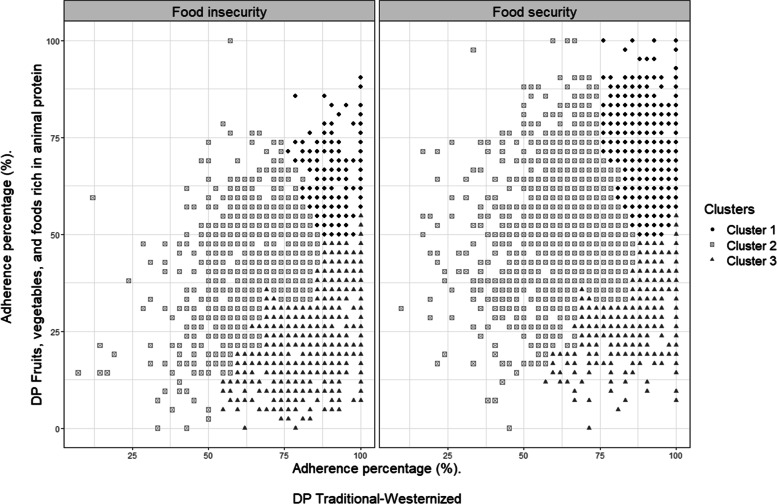
Scatter plot of adherence to dietary patterns by households’ food security and by households cluster. DP: Dietary Pattern. In the scatter plot black diamonds represent cluster 1 (*n* = 2247) including university students’ households with moderate adherence to the DP “Fruits, vegetables and foods rich in animal protein” (mean percentage of adherence 66.5 ± 10.2) and high adherence to DP “Traditional-Westernized” (mean percentage of adherence 91.3 ± 6.5); light grey squares represent cluster 2 (*n* = 3255) including university students’ households lacking adherence to the DP “Fruits, vegetables, and foods rich in animal protein” (mean percentage of adherence 49.2 ± 14) and moderate adherence to the DP “Traditional-Westernized” (mean percentage of adherence 68.1 ± 12.5); dark grey triangles represent cluster 3 (*n* = 2157) including university students’ households with non-adherence to the DP “Fruits, vegetables and foods rich in animal protein” (mean adherence 32.6 ± 11.7), and high adherence to the DP “Traditional-Westernized” (mean percentage adherence 88.5 ± 10.5). In this scatter plot the points also represent university students’ households with and without some degree of food insecurity. The household’s food insecurity category includes mild, moderate, and severe food insecurity

Figure 2 illustrates the adherence percentage to both DPs, differentiating between university students’ households with and without FI. In this graph, we can observe that most of those with some degree of FI have adherence (adherence percentage ≥ 51) to the DP T-W and no adherence (adherence percentage ≤ 50) to the DP FV-AP.

In addition, three groups were identified from the cluster analysis, as shown in Fig. 2. University students’ households grouped in cluster 1 (*n* = 2247) had moderate adherence to the DP FV-AP (mean adherence percentage 66.5 ± 10.2) and high adherence to DP T-W (mean adherence percentage 91.3 ± 6.5). In this cluster, 14.2% had FI (see Additional file 2). Cluster 2 (*n* = 3255) included university students’ households lacking adherence to the DP FV-AP (mean adherence percentage 49.2 ± 14) and moderate adherence to the DP T-W (mean adherence percentage 68.1 ± 12.5). In this cluster, 28.9% had FI (see Additional file 2). Cluster 3 (*n* = 2157) was characterized by including households whose inhabitants showed non-adherence to the DP FV-AP (mean adherence percentage 32.6 ± 11.7) and high adherence to DP T-W (mean adherence percentage 88.5 ± 10.5). This group showed the highest frequency of FI (50.9%) (see Additional file 2). The percentage adherence to each DP was significantly different between the three clusters. The characteristics of the households that constitute each cluster can be seen in the Additional file 2.

Discriminant analysis indicated that 98.2% (*n* = 2207) of the cases were correctly classified in cluster 1, 86.4% (*n* = 2811) in cluster 2, and 90.7% (*n* = 1956) in cluster 3. The overall classification of cases was 91.1%.

Table 4 shows the characteristics of the university students and their households according to adherence to the two DPs. Less than half of the households (47.7%) adhered to the DP FV-AP. Higher adherence to this DP was observed in those with food security (58.5%), high (67.8%) and medium–high (60.1%) socioeconomic status, highly educated (graduate or postgraduate) head of household (67.5%), and in those located in a metropolitan area (56.8%), among other characteristics. Moreover, more than 90% of household members, adhered to the DP T-W regardless of their personal, occupational, and sociodemographic characteristics. Significant differences were observed in the features of participants who did or did not adhere to this DP (Table 4).

**Table 4:** University students’ sociodemographic characteristics and household food security status according to adherence to dietary patterns

	Dietary Patterns					
	Fruits, vegetables, and foods rich in animal protein pattern			Traditional-Westernized pattern		
	Non-adherence (*n* = 4007; 52.3%)	Adherence (*n* = 3652; 47.7%)	*p* ^a^	Non-adherence (*n* = 331; 4.3%)	Adherence (*n* = 7328; 95.7%)	*p*^a^
*University students’ characteristics*						
Age (years), *mean (SD)*	21.9 (5.1)	22.5 (5.5)	< 0.001	22.8 (5.6)	22.2 (5.3)	0.049
Sex, *n (%)*			0.478			0.312
Man	1981 (52.7)	1775 (47.3)		153 (4.1)	3603 (95.9)	
Woman	2026 (51.9)	1877 (48.1)		178 (4.6)	3725 (95.4)	
Marital status			0.001			0.094
Not in a relationship (single, separated, divorced, or widower)	3643 (53)	3234 (47)		288 (4.2)	6589 (95.8)	
Partnered (living together, cohabiting or married)	364 (46.5)	418 (53.5)		43 (5.5)	739 (94.5)	
Enrollment college type			< 0.001			0.015
Private	1041 (48.3)	1115 (51.7)		113 (5.2)	2043 (94.8)	
Public	2966 (53.9)	2537 (46.1)		218 (4.0)	5285 (96.0)	
Academic year			0.001			0.051
3rd to 5th year	1601 (49.9)	1610 (50.1)		143 (4.5)	3068 (95.5)	
2nd year	1010 (54.0)	859 (46.0)		95 (5.1)	1774 (94.9)	
1st year	1396 (54.1)	1183 (45.9)		93 (3.6)	2486 (96.4)	
Scholarship student			0.474			0.646
No	3376 (52.5)	3054 (47.5)		275 (4.3)	6155 (95.7)	
Yes	631 (51.3)	598 (48.7)		56 (4.6)	1173 (95.4)	
Employment status in the month before the survey			0.086			0.648
No employed	2307 (51.5)	2171 (48.5)		189 (4.2)	4289 (95.8)	
Employed	1697 (53.5)	1474 (46.5)		141 (4.4)	3030 (95.6)	
Indigenous language			< 0.001			0.508
No	3912 (52.0)	3618 (48.0)		324 (4.3)	7206 (95.7)	
Yes	95 (73.6)	34 (26.4)		7 (5.4)	122 (94.6)	
Indigenous self-identification			< 0.001			0.093
No	2895 (48.9)	3027 (51.1)		243 (4.1)	5677 (95.9)	
Yes	1114 (64.1)	625 (35.9)		88 (5.1)	1651 (94.9)	
*University students’ household characteristics*						
Age of the household head (years), *mean (SD)*	48.7 (12.1)	48.6 (12.2)	0.821	41.9 (13.9)	48.9 (12.0)	< 0.001
Sex of the household head, *n (%)*			0.100			< 0.001
Man	2837 (52.9)	2522 (47.1)		194 (3.6)	5165 (96.4)	
Woman	1170 (50.9)	1130 (49.1)		137 (6.0)	2163 (94.0)	
Education of the household head			< 0.001			0.178
Bachelor´s degree or more	484 (32.5)	1003 (67.5)		67 (4.5)	1420 (95.5)	
Elementary to high school	3046 (55.5)	2446 (44.5)		244 (4.4)	5248 (95.6)	
Incomplete elementary school or less	477 (70.1)	203 (29.9)		20 (2.9)	660 (97.1)	
Household type			0.628			< 0.001
Nuclear	2664 (52.0)	2460 (48.0)		214 (4.2)	4910 (95.8)	
Extended	1167 (52.8)	1044 (47.2)		58 (2.6)	2153 (97.4)	
Others ^b^	176 (54.3)	148 (45.7)		59 (18.2)	265 (81.8)	
Children under 18-years-old			< 0.001			0.040
Yes	1398 (71.1)	569 (28.9)		69 (3.5)	1898 (96.5)	
No	2609 (45.8)	3083 (54.2)		262 (4.6)	5430 (95.4)	
Type of locality			< 0.001			< 0.001
Metropolitan (≥ 100,000 inhabitants)	1749 (43.2)	2304 (56.8)		210 (5.2)	3843 (94.8)	
Urban (≥ 2,500 to 99,999 inhabitants)	1160 (58.0)	839 (42.0)		78 (3.9)	1921 (96.1)	
Rural (< 2,500 inhabitants)	1098 (68.3)	509 (31.7)		43 (2.7)	1564 (97.3)	
Socioeconomic status			< 0.001			< 0.001
High	332 (32.2)	700 (67.8)		69 (6.7)	963 (93.3)	
Upper-middle	817 (39.9)	1231 (60.1)		95 (4.6)	1953 (95.4)	
Lower-middle	2279 (59.1)	1580 (40.9)		147 (3.8)	3712 (96.2)	
Low	579 (80.4)	141 (19.6)		20 (2.8)	700 (97.2)	
Household food security status ^c^			< 0.001			0.001
Food security	2198 (41.5)	3103 (58.5)		208 (3.9)	5093 (96.1)	
Mild food insecurity	884 (71.3)	356 (28.7)		53 (4.3)	1187 (95.7)	
Moderate food insecurity	550 (80.9)	130 (19.1)		36 (5.3)	644 (94.7)	
Severe food insecurity	375 (85.6)	63 (14.4)		34 (7.8)	404 (92.2)	

Data are presented as mean (standard deviation, SD) for continuous variables and frequency (percentage) for categorical variables

Adherence: percentage of adherence ≥ 51, Non-adherence: percentage of adherence ≤ 50

*P* < 0.05 was considered significant

^a^ *p*-Values from chi-square and Fisher’s exact tests or independent Student’s T-test, as appropriate

^b^ Others: single-person, composite and co-residential household

^c^ According to the Mexican Food Security Scale

### Association between household food insecurity and adherence to the dietary patterns 

Table 5 shows the associations between university students’ household food security status and adherence to the two identified DPs. A graded inverse association was observed between the degree of FI at household with adherence to the DP FV-AP (mild-FI OR: 0.34, 95%CI: 0.30, 0.40, *p* < 0.001; moderate-FI OR: 0.20, 95%CI: 0.16, 0.24, *p* < 0.001; severe-FI OR: 0.14, 95%CI: 0.11, 0.19, *p* < 0.001), after complete adjustments for potential confounders, compared to university students’ household food security. Further, compared with food-secure households, those with severe-FI were significantly less likely to adhere to DP T-W after adjustment (OR: 0.51, 95%CI: 0.34, 0.76, *p* = 0.001) (Table 5).

**Table 5:** Association between household food insecurity status^a^ and adherence to the two identified dietary patterns

Adherence to dietary patterns	Food security (*n* = 5301)	Mild-Food Insecurity (*n* = 1240)	*p*^b^	Moderate-Food Insecurity (*n* = 680)	*p*	Severe-Food Insecurity (*n* = 438)	*p*^b^
	OR (95% CI)	OR (95% CI)		OR (95% CI)		OR (95% CI)	
Fruits, vegetables, and foods rich in animal protein pattern							
Crude	1.00	0.29 (0.25, 0.33)	< 0.001	0.17 (0.14, 0.20)	< 0.001	0.12 (0.09, 0.16)	< 0.001
Model 1	1.00	0.30 (0.26, 0.34)	< 0.001	0.18 (0.15, 0.22)	< 0.001	0.13 (0.10, 0.17)	< 0.001
Model 2	1.00	0.34 (0.30, 0.40)	< 0.001	0.20 (0.16, 0.24)	< 0.001	0.14 (0.11, 0.19)	< 0.001
Traditional-Westernized pattern							
Crude	1.00	0.91 (0.67, 1.24)	0.570	0.73 (0.51, 1.05)	0.090	0.49 (0.33, 0.71)	< 0.001
Model 1	1.00	0.91 (0.67, 1.24)	0.557	0.74 (0.51, 1.06)	0.101	0.48 (0.33, 0.70)	< 0.001
Model 2	1.00	0.82 (0.60, 1.13)	0.225	0.69 (0.47, 1.00)	0.053	0.51 (0.34, 0.76)	0.001

Data are presented as Odds Ratio (OR) with correspondent 95% confidence interval (95% CI)

Model 1: adjusted for students’ sex (man, woman), age (years), marital status (not in a relationship, partnered), indigenous self-identification (no, yes), academic year (3rd to 5th year, 2nd year, 1st year), and enrollment college type (private, public)

Model 2: adjusted by model 1 variables plus household’s head age (years), household’s head sex (man, woman), household’s head education (bachelor´s degree or more, elementary to high school, incomplete elementary school or less); household type (nuclear, extended, others), type of locality (metropolitan, urban, rural), and socioeconomic status (high, upper-middle, lower-middle, low)

*p* < 0.05 was considered significant

Adherence: percentage of adherence ≥ 51, Non-adherence: percentage of adherence ≤ 50

^a^ According to the Mexican Food Security Scale

^b^*p*-Values from logistic regression

## Discussion

This work is the first study to analyze the association between FI and DPs, generated by multivariate analysis, in households of university students. We identified two DPs; the first was labeled “Fruits, vegetables, and foods rich in animal protein” (DP FV-AP) and consisted of fruits, vegetables, meat, fish, dairy products, and starchy vegetables. The second DP, labeled “Traditional-Westernized” (DP T-W), consisted of pulses, oils, sugars, cereals, eggs, condiments, coffee, and tea. Our results demonstrate that compared with food-secure households, those with any degree of FI were associated with decreased odds of adhering to the DP FV-AP after multivariate adjustment. Additionally, those with severe FI were less likely to stick to the DP T-W. Furthermore, from three groups/clusters of households analyzed according to each DP’s adherence percentage, we observed the highest frequency of FI amongst the cluster including those who did not adhere to the DP FV-AP and had high adherence to the DP T-W.

Few studies have examined the association between FI and DPs through principal components analysis [19–23]. In line with our findings, a cross-sectional survey among 406 schools with 31 399 students (16 652 children and 14 747 adolescents), conducted in Greece, showed that mild or severe FI in children was negatively associated with both a DP consisting of fruits, vegetables, natural fruit juice, and whole grains and a DP consisting of foods rich in animal protein (red meat, poultry, fish) [19]. Also, in adolescents, mild or severe FI was negatively associated with a DP consisting of natural fruit juice and fruits and was also negatively associated with a DP consisting of red meat, poultry, fish, and traditional meat dishes [19]. Similarly, other cross-sectional studies also found a significant inverse association between moderate and severe FI in adults from two ethnic groups [21], higher FI scores in adolescents [22], and the presence of FI in people older than 15 years [23] with adherence to DPs including healthy food groups such as fruits [21–23], vegetables [21, 22], natural fruit juice [21], wholegrain cereals [22], olives and nuts [21, 22], olive oil [22], pulses [22], dairy products [21, 22, 37], tea [23], and soy and rice milk [23]. None of these studies assessed the university students’ household food security status like the current study.

Other previous cross-sectional studies have identified “Traditional” DPs [19–21, 23] and unhealthy DPs [19–21] and associated them with FI. In contrast to our results, FI has been positively associated with “Traditional” DP [19–21, 23]. However, foods considered in each type of traditional DP in the different studies were diverse, depending on the origin of the population studied. For instance, some foods that have been included in this DP are traditionally vegetarian and meat dishes [19, 21], pulses [19–21], eggs [19, 21], tea [21, 23], sugar [20, 23], sauces [20, 23], fats [20, 21] and cereals (rice, bread, whole grain) [20, 21]. Several foods that constituted the “Traditional” DP were similar to those in our DP T-W. Nevertheless, in our analysis, a negative association was observed between severe FI and the DP T-W, and no significant association was observed between mild and moderate FI and this DP. However, in the cluster with the highest frequency of any degree of FI, notably adherence to the DP T-W was observed.

On the other hand, other studies have found FI to be negatively associated with unhealthy DP, labeled “Transitional” [21] or “Western” DP [20]. Like our DP T-W, this DP comprises sweetened drinks [20, 21], and sweets and confectionery [20, 21]. This unhealthy DP also included fast foods [20, 21], red meat and organic meat [21], savory snacks [20, 21], sauces (ketchup and mayonnaise), poultry, fish and shrimp, seeds and coffee [21], cakes and cookies, milk and dairy products [20]. In contrast, in children, FI with or without hunger has been positively associated with a DP that included chips, fast-food, sugary drinks, sweets, French fries and mayonnaise sauce [19].

People with FI often develop specific coping strategies to access food [10, 13, 16]. Students with severe FI tend to use more coping strategies to acquire food [13, 16]. These coping strategies may explain why, in this study, members of households with FI were less likely to adhere to a DP consisting of healthy foods, such as fruit and vegetables, roots, and starchy vegetables, as well as protein-rich foods, such as meat, fish, and dairy products. These coping strategies could also explain why the cluster with the higher frequency of FI showed higher adherence to the DP T-W and why households with extreme FI were less likely to adhere to both DPs. Some of the coping strategies that university students [10, 13] and families with FI in general [16, 38] have used are: 1.- buying the cheapest food available even if it is not the healthiest [10, 38, 39], 2.- purchasing and consuming cheap and processed foods (frozen pizza, sweets) [13, 16], 3.- eating less nutritious meals to consume more food [13, 16], 4.- buying foods that do not spoil quickly (such as pasta, beans, rice, canned foods), and 5.- stretching food by eating less so it lasts longer [13, 16, 38, 39].

In addition, one of the main reasons many people cannot access a healthy diet, including foods from several food groups and with greater variety, is its higher cost [2, 40–42]. University students with FI report that one of the barriers to accessing food more frequently (very often/often) compared to food-secure students is the high cost of food [10]. For instance, in a sample of Australian university students, the majority were dissatisfied with the cost of food and drinks and one of the main changes suggested in the campus food environment was cheaper food [43]. The cost of the diet increases as the quality of the diet increases. In 2017, the global average price of a healthy diet was 60% higher than a nutritionally adequate diet and almost five times higher than the cost of an energy-sufficient diet. In this period, a nutritionally adequate diet cost USD 2.33 per person per day, while a healthy diet cost USD 3.75 per day [40]. In the same year, the global average cost of nutritious foods such as fruits, vegetables, and foods of animal origin (all types of meat, poultry, fish, eggs, milk, cheese, yogurt and other dairy products), was higher than that of foods high in fat, sugar or salt and higher than that of foods containing starch, oils, and sugars. However, these costs vary from country to country. Especially in lower-income countries, most foods of animal origin tend to be more expensive. Starchy foods and oils account for 20% of the cost of a healthy diet. Fruit and vegetables account for just under 40% of their price, and dairy products and protein foods just over 40% [40]. Therefore, the cost of healthy foods is a crucial determinant of dietary choices [39, 40, 42].

Low income is another primary reason for not accessing a healthy diet [2, 39–41]. In the present study, 59.8% of surveyed participants had a low or lower-middle socioeconomic level. People on lower incomes may be limited in purchasing healthy foods and meeting their basic needs [40, 41, 44]. Even having an economic restriction when selecting their food choices can lead to deficient animal protein diets [41]. In addition, low-income neighborhoods often have less access to healthy foods. They have a smaller selection of supermarkets, which tend to offer less variety of fresh products and lower quality products. To access healthy food options, this population often needs to purchase them outside their area of residence. Still, this access may be limited by the cost of transport, lack of access to public transportation, or the time available to shop for these foods [41].

The unhealthy food environment with a higher frequency of street vendors and shops selling junk food [41, 42] is another barrier to accessing healthier food options. Particularly, regarding the food environment in universities, one study showed that most college students were dissatisfied with the types of main meals and snacks available on campus. The main changes these students suggested included healthier options and higher-quality foods [43]. Another study that evaluated the characteristics of the food sales establishments located inside and outside of university centers in Mexico identified that none offered the nutritional value of the dishes, only 20.3% of the establishments evaluated had a salad bar, 46.2% provided preparations with vegetables, and 51.9% offered sugar-free drinks. Additionally, 28% were street vendors. The drink that was offered in a greater number of establishments was soft drinks, and 84% of the establishments sold one or more ultra-processed foods [45]. Other barriers may include difficulties in transport to and from the food shops, prioritizing the price of food in food selection, often sacrificing food quality [39, 41], limited time to buy and prepare food, or even not having access to a kitchen or having inadequate spaces to store food [41].

Finally, food preference is another crucial determinant of adherence to a DP. While it is true that those with some degree of FI are less likely to follow a DP that includes fruits and vegetables, regardless of the degree of household food security, less than half of the households in the present study consume fruit and vegetables daily. Also, over half consume sugar, sweets, soft drinks, or industrialized beverages daily. That is, in this study population, there was no preference for the consumption of fruits and vegetables, and there was a preference for the consumption of sugars, sweets, and sugary drinks. Why not replace the purchase of sugars and sugary drinks with the purchase of fruits and vegetables? Similarly, in a study of college students, fruit and vegetable intake was low, while the average intake of added sugars was high [7].

Addressing the FI problem requires social programs focused primarily on improving education and food self-sufficiency. Education would allow for better job opportunities or, in general, better skills and abilities to generate higher levels of income [44]. Increasing the availability of safe and nutritious food while reducing its cost, food losses and waste, requires policies, investments, and legislation, from production to consumption [2, 46]. It is also necessary to create healthier food environments, and promote a nutritious, healthy, and safe diet that has a less negative impact on the environment [2, 40, 42, 46]. Interventions should be country-specific and account for consumption habits [2].

Among other relevant results, only 47.7% showed adherence to “Fruits, vegetables, and foods rich in animal protein pattern”. Adherence to this DP, is appropriate because a low intake of fruits and vegetables is associated with a higher risk of cardiovascular disease, cancer, and all-cause mortality [47]. Other foods that make up the DP FV-AP include meat, dairy products, and fish. The daily consumption of red meat is not recommended because it increases health risks, such as type 2 diabetes [48] and cardiovascular disease [49]. Therefore participants were expected to have less than 100% adherence to this DP. Otherwise, consuming dairy products and fish is negatively associated with type 2 diabetes [48] and a lower risk of stroke [50] respectively. The DPs of university students are relevant because metabolic syndrome components are already evident in this population, 53.7% of Mexican university students presented one or more metabolic syndrome components [51].

The main strengths of our study are the extension and the robust sampling method of the *ENIGH 2018* that allowed us to analyze households from all states in Mexico and with different sociodemographic characteristics, as well as the use of a validated scale to examine the degree of food security/insecurity (*EMSA*) (29). Nevertheless, given the cross-sectional nature of our study, causality cannot be assumed, and the results presented must be interpreted with caution. The *ENIGH* 2018 database contains information on food consumption concerning all household members and not exclusively for university students. In addition, each food group that constitutes the consumption frequency questionnaire includes a wide variety of foods which did not allow the generation of detailed DPs. These limitations also favor that DPs do not differ between different population groups. In this sense, we analyzed DPs in households without college students and identified similar DPs (see Additional file 3). Although there is a possibility that DPs are similar in homes with and without college students since 95.8% of these students lived with their family members. Nevertheless, analyzing whether there are differences in the DPs of university students and other population groups is an interesting research question to answer in futures studies. Otherwise, only foods that were prepared within the home were considered. Foods that were purchased ready-made to be consumed at home were not considered. In addition, several physiological, cultural, social, and personal factors also determine adherence to certain DPs and are not considered in the present analysis, like the presence of some illnesses. Future studies must identify the determinants of food consumption in those who have or do not have FI. However, despite the limitations of this study, this analysis is the first approach considering the scarce evidence in this population group.

We suggest analyzing the association between FI and DPs based on more detailed information on food consumption only in university students. In addition, we recommend performing this analysis at the current time because, during the COVID-19 pandemic, the frequency of FI increased worldwide [2]. Further, global food prices have risen sharply due to supply chain disruptions and the repercussions of war and pandemics. The most affected products are wheat, corn, edible oils, and fertilizers [52]. So, the DPs could change in this and other population groups.

## Conclusion

In this study, university students’ household with food insecurity status was associated with decreased odds of adhering to a DP characterized by fruits, vegetables, meat, fish, dairy products, roots, and starchy vegetables, labeled “Fruits, vegetables, and foods rich in animal protein.” In addition, those with severe FI had a lower likelihood of adhering to a DP characterized by pulses, seeds, oils, fats, sugar, sweets, industrialized beverages, foods made from corn, wheat, rice, oats or bran, eggs, condiments, coffee, and tea, labeled “Traditional-Westernized”. Recognizing the less healthy DPs in households with varying degrees of FI can enable strategies that promote healthy food groups which are less represented and more available or accessible, thus, contributing to better-quality diets.

## Supplementary Information

**Additional file 1. **Factor loadings for the two factors/dietary patterns derived from 12 food groups. This table shows, in bold, the food groups that constitute the dietary pattern “Fruits, vegetables, and food rich in animal protein” and the dietary pattern “Traditional-Westernized”.

**Additional file 2. **Sociodemographic characteristics by cluster. This table shows the university students’ characteristics and university students’ household characteristics by cluster.

**Additional file 3. **Factor loading for the two factors/dietary patterns derived from 12 food groups in households without college students. This table shows, in bold, the food groups that constitute the dietary pattern “Fruits, vegetables, and food rich in animal protein” and the dietary pattern “Traditional-Westernized” in households without college students.

## Data Availability

The datasets analysed during the current study are available in the *Encuesta Nacional de Ingresos y Gastos de los Hogares* (*ENIGH*). 2018 Nueva serie: https://www.inegi.org.mx/programas/enigh/nc/2018/#Datos_abiertos [30]

## Abbreviations

FI: Food insecurity
DP: Dietary pattern
DPs: Dietary patterns

## References

[1] Organización de las Naciones Unidas para la Alimentación y la Agricultura (2021). Hambre e inseguridad alimentaria.

[2] FAO, FIDA, OMS, PMA, UNICEF. El estado de la seguridad alimentaria y la nutrición en el mundo 2021. Transformación de los sistemas alimentarios en aras de la seguridad alimentaria, una nutrición mejorada y dietas asequibles y saludables para todos. Roma; 2021.

[3] Nikolaus CJ, An R, Ellison B, Nickols-Richardson SM (2020). Food insecurity among college students in the United States: a scoping review. Adv Nutr.

[4] Bruening M, Argo K, Payne-Sturges D, Laska MN (2017). The struggle is real: a systematic review of food insecurity on postsecondary education campuses. J Acad Nutr Diet.

[5] Sidebottom C, Ullevig S, Cheever K, Zhang T (2021). Effects of COVID-19 pandemic and quarantine period on physical activity and dietary habits of college-aged students. Sport Med Heal Sci.

[6] Mialki K, House LA, Mathews AE, Shelnutt KP (2021). Covid-19 and college students: Food security status before and after the onset of a pandemic. Nutrients.

[7] Leung CW, Wolfson JA, Lahne J, Barry MR, Kasper N, Cohen AJ (2019). Associations between food security status and diet-related outcomes among students at a large, public Midwestern university. J Acad Nutr Diet.

[8] Nava-Amante P, Betancourt-Núñez A, Vizmanos B, Salas-García M, Bernal-Orozco M, Vargas-García E (2021). Prevalence and risk factors of food insecurity among Mexican university students’ households. Nutrients.

[9] El Zein A, Colby SE, Zhou W, Shelnutt KP, Greene GW, Horacek TM (2020). Food insecurity is associated with increased risk of obesity in US college students. Curr Dev Nutr.

[10] Hiller MB, Winham DM, Knoblauch ST, Shelley MC (2021). Food security characteristics vary for undergraduate and graduate students at a Midwest University. Int J Environ Res Public Health.

[11] Laska MN, Lenk K, Lust K, McGuire CM, Porta CM, Stebleton M (2020). Sociodemographic and health disparities among students screening positive for food insecurity: Findings from a large college health surveillance system. Prev Med Reports.

[12] El Zein A, Shelnutt KP, Colby S, Vilaro MJ, Zhou W, Greene G (2019). Prevalence and correlates of food insecurity among U.S. college students: A multi-institutional study. BMC Public Health.

[13] McArthur LH, Ball L, Danek AC, Holbert D (2018). A high prevalence of food insecurity among university students in Appalachia reflects a need for educational interventions and policy advocacy. J Nutr Educ Behav.

[14] Martinez SM, Grandner MA, Nazmi A, Canedo ER, Ritchie LD (2019). Pathways from food insecurity to health outcomes among California university students. Nutrients.

[15] Christensen KA, Forbush KT, Richson BN, Thomeczek ML, Perko VL, Bjorlie K (2021). Food insecurity associated with elevated eating disorder symptoms, impairment, and eating disorder diagnoses in an American University student sample before and during the beginning of the COVID-19 pandemic. Int J Eat Disord.

[16] McArthur LH, Fasczewski KS, Wartinger E, Miller J (2018). Freshmen at a University in Appalachia experience a higher rate of campus than family food insecurity. J Community Health.

[17] Shi Y, Davies A, Allman-Farinelli M (2021). The association between food insecurity and dietary outcomes in university students: a systematic review. J Acad Nutr Diet.

[18] Theodoridis X, Grammatikopoulou MG, Gkiouras K, Papadopoulou SE, Agorastou T, Gkika I (2018). Food insecurity and Mediterranean diet adherence among Greek university students. Nutr Metab Cardiovasc Dis.

[19] Kastorini CM, Markaki I, Tsiampalis T, Critselis E, Petralias A, Linos A (2021). Dietary patterns and food insecurity of students participating in a food aid programme: the Mediterranean perspective. Eur J Public Health.

[20] Cunha DB, Sichieri R, De Almeida RMVR, Pereira RA (2011). Factors associated with dietary patterns among low-income adults. Public Health Nutr.

[21] Rezazadeh A, Omidvar N, Eini-Zinab H, Ghazi-Tabatabaie M, Majdzadeh R, Ghavamzadeh S (2016). Major dietary patterns in relation to demographic and socio-economic status and food insecurity in two Iranian ethnic groups living in Urmia. Iran Public Health Nutr.

[22] Naja F, Itani L, Kharroubi S, Diab El Harake M, Hwalla N, Jomaa L (2020). Food insecurity is associated with lower adherence to the Mediterranean dietary pattern among Lebanese adolescents: a cross-sectional national study. Eur J Nutr.

[23] Beck KL, Jones B, Ullah I, McNaughton SA, Haslett SJ, Stonehouse W (2018). Associations between dietary patterns, socio-demographic factors and anthropometric measurements in adult New Zealanders: an analysis of data from the 2008/09 New Zealand Adult Nutrition Survey. Eur J Nutr.

[24] Hu FB (2002). Dietary pattern analysis: a new direction in nutritional epidemiology. Curr Opin Lipidol.

[25] Arnett JJ (2000). Emerging adulthood. A theory of development from the late teens through the twenties. Am Psychol.

[26] Arnett JJ (2016). College students as emerging adults: the developmental implications of the college context. Emerg Adulthood.

[27] Ramón-Arbués E, Gea-Caballero V, Granada-López JM, Juárez-Vela R, Pellicer-García B, Antón-Solanas I (2020). The prevalence of depression, anxiety and stress and their associated factors in college students. Int J Environ Res Public Health.

[28] Castro O, Bennie J, Vergeer I, Bosselut G, Biddle SJH (2020). How sedentary are university students? A systematic review and meta-analysis. Prev Sci Off J Soc Prev Res.

[29] Hartmann ME, Prichard JR (2018). Calculating the contribution of sleep problems to undergraduates’ academic success. Sleep Heal.

[30] Instituto Nacional de Estadística y Geografía. INEGI. Encuesta Nacional de Ingresos y Gastos de los Hogares (ENIGH). 2018 Nueva serie. 2019. https://www.inegi.org.mx/programas/enigh/nc/2018/#Datos_abiertos. Accessed 5 Jan 2021.

[31] Instituto Nacional de Estadística y Geografía. Encuesta Nacional de Ingresos y Gastos de los Hogares (ENIGH). 2018 Nueva serie. 2019. https://www.inegi.org.mx/programas/enigh/nc/2018/. Accessed 9 Feb 2021.

[32] Instituto Nacional de Estadística y Geografía. INEGI. Encuesta Nacional de Ingresos y Gastos de los Hogares. ENIGH 2018. Manual del Entrevistador. 2019.

[33] Instituto Nacional de Estadística y Geografía. INEGI. Encuesta Nacional de Ingresos y Gastos de los Hogares (ENIGH) 2018. Nueva Serie. Descripción de base de datos. 2019. https://www.inegi.org.mx/contenidos/programas/enigh/nc/2018/doc/enigh18_descriptor_archivos_fd_ns.pdf.

[34] Villagómez-Ornelas P, Hernández-López P, Carrasco-Enríquez B, Barrios-Sánchez K, Pérez-Escamilla R, Melgar-Quiñónez H (2014). Validez estadística de la Escala Mexicana de Seguridad Alimentaria y la Escala Latinoamericana y Caribeña de Seguridad Alimentaria. Salud Publica Mex.

[35] Sánchez-Villegas A, Bes-Rastrollo M, Martínez-González M. Análisis factorial. In: Martínez González M, Sánchez-Villegas A, Toledo Atucha E, Faulin Fajardo J, editors. Bioestadística Amigable. 3a edición. Barcelona: Elsevier; 2014. p. 487–510.

[36] Costello A, Osborne J (2005). Best practices in exploratory factor analysis: four recommendations for getting the most from your analysis. Pract Assess Res Eval.

[37] da Silva AAM, Simões VMF, Barbieri MA, Cardoso VC, Alves CMC, Thomaz EBAF (2014). A protocol to identify non-classical risk factors for preterm births: the Brazilian Ribeirão Preto and São Luís prenatal cohort (BRISA). Reprod Health.

[38] Niles MT, Bertmann F, Belarmino EH, Wentworth T, Biehl E, Neff R (2020). The early food insecurity impacts of COVID-19. Nutrients.

[39] Puddephatt JA, Keenan GS, Fielden A, Reaves DL, Halford JCG, Hardman CA (2020). “Eating to survive”: A qualitative analysis of factors influencing food choice and eating behaviour in a food-insecure population. Appetite.

[40] FAO, FIDA, OMS, PMA, UNICEF (2020). El estado de la seguridad alimentaria y la nutrición en el mundo 2020.

[41] Vilar-Compte M, Burrola-Méndez S, Lozano-Marrufo A, Ferré-Eguiluz I, Flores D, Gaitán-Rossi P (2021). Urban poverty and nutrition challenges associated with accessibility to a healthy diet: a global systematic literature review. Int J Equity Health.

[42] Herforth A, Ahmed S (2015). The food environment, its effects on dietary consumption, and potential for measurement within agriculture-nutrition interventions. Food Secur.

[43] Hutchesson MJ, Whatnall MC, Patterson AJ (2022). On-campus food purchasing behaviours and satisfaction of Australian university students. Heal Promot J Aust.

[44] Díaz-Carreño MA, Sánchez-Cándido LV, Herrera Rendón-Nebel MT. La inseguridad alimentaria severa en los estados de México: Un análisis a partir del enfoque de las capacidades 2008–2014. Estud Soc Rev Aliment Contemp y Desarro Reg. 2019;29:1–24.

[45] Gastelum Strozzi V, Márquez-Sandoval YF (2021). Disponibilidad y costo de alimentos ofertados dentro y fuera de los Centros Universitarios de la Universidad de Guadalajara de la Zona Metropolitana.

[46] Amorim A, de Holanda Barbosa A, do Amaral Sobral PJ (2022). Hunger, obesity, public policies, and food-based dietary guidelines: a reflection considering the socio-environmental world context. Front Nutr.

[47] Aune D, Giovannucci E, Boffetta P, Fadnes LT, Keum NN, Norat T (2017). Fruit and vegetable intake and the risk of cardiovascular disease, total cancer and all-cause mortality-a systematic review and dose-response meta-analysis of prospective studies. Int J Epidemiol.

[48] Tian S, Xu Q, Jiang R, Han T, Sun C, Na L (2017). Dietary protein consumption and the risk of type 2 diabetes: a systematic review and meta-analysis of cohort studies. Nutrients.

[49] Zhong VW, Van Horn L, Greenland P, Carnethon MR, Ning H, Wilkins JT (2020). Associations of processed meat, unprocessed red meat, poultry, or fish intake with incident cardiovascular disease and all-cause mortality. JAMA Intern Med.

[50] Chen C, Huang H, Dai QQ, Ren J, Cai HH, Hu WJ (2021). Fish consumption, long-chain omega-3 fatty acids intake and risk of stroke: An updated systematic review and meta-analysis. Asia Pac J Clin Nutr.

[51] Betancourt-Núñez A, Márquez-Sandoval F, Babio N, Vizmanos B (2018). Metabolic syndrome components in young health professionals; LATIN America METabolic Syndrome (LATINMETS) Mexico study. Nutr Hosp.

[52] Banco mundial. Actualización sobre la seguridad alimentaria. 2022. https://www.bancomundial.org/es/topic/agriculture/brief/food-security-update. Accessed 20 Aug 2022.

