# Supplementary material for: Food insecurity was negatively associated with adherence to the “fruits, vegetables, and foods rich in animal protein” dietary pattern among university students’ households: the 2018 Mexican National Household Survey
Source: BMC Public Health. 2023 May 11;23:854. doi: 10.1186/s12889-023-15755-z (PMC10208201; doi:10.1186/s12889-023-15755-z)
Supplement: Supplementary file 1 — Additional file 1. Factor loadings for the two factors/dietary patterns derived from 12 food groups. This table shows, in bold, the food groups that constitute the dietary pattern “Fruits, vegetables, and food rich in animal protein” and the dietary pattern “Traditional-Westernized”. [file 12889_2023_15755_MOESM1_ESM.docx]

**Additional file 1.** Factor loadings for the two factors**/**dietary patterns derived from 12 food groups.

| Food groups | “Fruits, vegetables, and foods rich in animal protein” pattern | “Traditional-westernized” pattern |
| --- | --- | --- |
| Fruits | **0.706** | 0.007 |
| Vegetables | **0.655** | 0.103 |
| Meat | **0.630** | -0.051 |
| Fish or seafood | **0.557** | -0.099 |
| Dairy products | **0.514** | 0.246 |
| Roots or starchy vegetables | **0.471** | 0.150 |
| Pulses or seeds | -0.017 | **0.590** |
| Oils or fats | 0.052 | **0.581** |
| Sugar, sweets, soft drinks or industrialized beverages | 0.021 | **0.581** |
| Foods made from corn, wheat, rice, oats or bran. | -0.100 | **0.540** |
| Condiments, coffee or tea | 0.135 | **0.464** |
| Eggs | 0.191 | **0.434** |
| Variance (%) | 19.70 | 13.75 |

Principal Component Analysis.

Factor groups with factor load ≥0.30 were considered the major foods associated with the dietary pattern and are marked in bold**.**

This table shows, in bold, the food groups that constitute the dietary pattern “Fruits, vegetables, and food rich in animal protein” and the dietary pattern “Traditional-westernized”.
