# Supplementary material for: Food insecurity was negatively associated with adherence to the “fruits, vegetables, and foods rich in animal protein” dietary pattern among university students’ households: the 2018 Mexican National Household Survey
Source: BMC Public Health. 2023 May 11;23:854. doi: 10.1186/s12889-023-15755-z (PMC10208201; doi:10.1186/s12889-023-15755-z)
Supplement: Supplementary file 2 — Additional file 2. Sociodemographic characteristics by cluster. This table shows the university students’ characteristics and university students’ household characteristics by cluster. [file 12889_2023_15755_MOESM2_ESM.docx]

**Additional file 2**. Sociodemographic characteristics by cluster

|  | Cluster 1 | Cluster 2 | Cluster 3 | P ^a^ |
| --- | --- | --- | --- | --- |
|  | n (%) | n (%) | n (%) |  |
| *University students’ characteristics* | |  |  |  |
| Sex |  |  |  | 0.281 |
| Man | 1092 (48.6) | 1575 (48.4) | 1089 (50.5) |  |
| Woman | 1155 (51.4) | 1680 (51.6) | 1068 (49.5) |  |
| Marital status |  |  |  | <0.001 |
| Partnered (living together, cohabiting or married) | 240 (10.7) | 384 (11.8) | 158 (7.3) |  |
| Not in a relationship (single, separated, divorced, or widower) | 2007 (89.3) | 2871 (88.2) | 1999 (92.7) |  |
| Enrollment college type |  |  |  | <0.001 |
| Public | 1568 (69.8) | 2304 (70.8) | 1631 (75.6) |  |
| Private | 679 (30.2) | 951 (29.2) | 526 (24.4) |  |
| Academic year |  |  |  | 0.001 |
| 1st year | 726 (32.3) | 1079 (33.1) | 774 (35.9) |  |
| 2nd year | 514 (22.9) | 796 (24.5) | 559 (25.9) |  |
| 3rd to 5th year | 1007 (44.8) | 1380 (42.4) | 824 (38.2) |  |
| Scholarship student |  |  |  | 0.394 |
| Yes | 355 (15.8) | 543 (16.7) | 331 (15.3) |  |
| No | 1892 (84.2) | 2712 (83.3) | 1826 (84.7) |  |
| Employment status in the month before the survey |  |  |  | 0.100 |
| Not employed | 891 (39.7) | 1386 (42.6) | 894 (41.4) |  |
| Employed | 1351 (60.3) | 1864 (57.4) | 1263 (58.6) |  |
| Indigenous language |  |  |  | <0.001 |
| Yes | 20 (0.9) | 52 (1.6) | 57 (2.6) |  |
| No | 2227 (99.1) | 3203 (98.4) | 2100 (97.4) |  |
| Indigenous self-identification |  |  |  | <0.001 |
| Yes | 360 (16.0) | 774 (23.8) | 605 (28.0) |  |
| No | 1887 (84.0) | 2481 (76.2) | 1552 (72.0) |  |
| Sex of the household head |  |  |  | <0.001 |
| Man | 1602 (71.3) | 2196 (67.5) | 1561 (72.4) |  |
| Woman | 645 (28.7) | 1059 (32.5) | 596 (27.6) |  |
| *University students’ household characteristics* | |  |  |  |
| Education of the household head |  |  |  | <0.001 |
| Incomplete elementary school or less | 144 (6.4) | 231 (7.1) | 305 (14.1) |  |
| Elementary to high school | 1517 (67.5) | 2313 (71.1) | 1662 (77.1) |  |
| Bachelor´s degree or more | 586 (26.1) | 711 (21.8) | 190 (8.8) |  |
| Household type |  |  |  | <0.001 |
| Nuclear | 1488 (66.2) | 2215 (68.0) | 1421 (65.9) |  |
| Extended | 700 (31.2) | 816 (25.1) | 695 (32.2) |  |
| Other ^b^ | 59 (2.6) | 224 (6.9) | 41 (1.9) |  |
| Children under 18-years-old |  |  |  | <0.001 |
| Yes | 347 (15.4) | 749 (23.0) | 871 (40.4) |  |
| No | 1900 (84.6) | 2506 (77.0) | 1286 (59.6) |  |
| Type of locality |  |  |  | <0.001 |
| Metropolitan (≥100,000 inhabitants) | 1400 (62.3) | 1814 (55.7) | 839 (38.9) |  |
| Urban (≥2,500 to 99,999 inhabitants) | 527 (23.5) | 832 (25.6) | 640 (29.7) |  |
| Rural (< 2,500 inhabitants) | 320 (14.2) | 609 (18.7) | 678 (31.4) |  |
| Socioeconomic status |  |  |  | <0.001 |
| Low | 89 (4.0) | 252 (7.7) | 379 (17.6) |  |
| Lower-middle | 987 (43.9) | 1607 (49.4) | 1265 (58.6) |  |
| Upper-middle | 753 (33.5) | 893 (27.4) | 402 (18.6) |  |
| High | 418 (18.6) | 503 (15.5) | 111 (5.1) |  |
| Household food security status ^c^ |  |  |  | <0.001 |
| Food security | 1929 (85.8) | 2313 (71.1) | 1059 (49.1) |  |
| Mild food insecurity | 205 (9.1) | 532 (16.3) | 503 (23.3) |  |
| Moderate food insecurity | 78 (3.5) | 252 (7.7) | 350 (16.2) |  |
| Severe food insecurity | 35 (1.6) | 158 (4.9) | 245 (11.4) |  |
| Percentage adherence to the DP “Fruits, vegetables and foods rich in animal protein” |  |  |  | <0.001 |
| ≤50 | 87 (3.9) | 1849 (56.8) | 2071 (96.0) |  |
| 51 a 75 | 1715 (76.3) | 1304 (40.1) | 86 (4.0) |  |
| 76 a 100 | 445 (19.8) | 102 (3.1) | 0 (0) |  |
| Percentage adherence to the DP “Basic food basket and sugars” |  |  |  | <0.001 |
| ≤50 | 0 (0) | 331 (10.2) | 0 (0) |  |
| 51 a 75 | 0 (0) | 1781 (54.7) | 316 (14.6) |  |
| 76 a 100 | 2247 (100) | 1143 (35.1) | 1841 (85.4) |  |

^a^ *p* values from chi-square tests

**^b^** Other: single-person, composite and co-residential household.

**^c^** According to the Mexican Food Security Scale.

Data are presented as frequency and percentage.

This table shows the university students’ characteristics and university students’ household characteristics by cluster.
