# Supplementary material for: Food insecurity was negatively associated with adherence to the “fruits, vegetables, and foods rich in animal protein” dietary pattern among university students’ households: the 2018 Mexican National Household Survey
Source: BMC Public Health. 2023 May 11;23:854. doi: 10.1186/s12889-023-15755-z (PMC10208201; doi:10.1186/s12889-023-15755-z)
Supplement: Supplementary file 3 — Additional file 3. Factor loading for the two factors/dietary patterns derived from 12 food groups in households without college students. This table shows, in bold, the food groups that constitute the dietary pattern “Fruits, vegetables, and food rich in animal protein” and the dietary pattern “Traditional-Westernized” in households without college students. [file 12889_2023_15755_MOESM3_ESM.docx]

**Additional file 3.** Factor loadings for the two factors**/**dietary patterns derived from 12 food groups in households without college students

| Food groups | “Fruits, vegetables, and foods rich in animal protein” pattern | “Traditional-Westernized” pattern |
| --- | --- | --- |
| Fruits | **0.700** | -0.018 |
| Vegetables | **0.665** | 0.071 |
| Meat | **0.662** | -0.036 |
| Dairy products | **0.577** | 0.214 |
| Fish or seafood | **0.502** | -0.081 |
| Roots or starchy vegetables | **0.468** | 0.156 |
| Sugar, sweets, soft drinks or industrialized beverages | 0.045 | **0.623** |
| Oils or fats | 0.116 | **0.550** |
| Pulses or seeds | -0.032 | **0.549** |
| Condiments, coffee or tea | 0.049 | **0.548** |
| Foods made from corn, wheat, rice, oats or bran. | -0.080 | **0.446** |
| Eggs | 0.230 | **0.429** |

Principal Component Analysis.

Factor groups with factor load ≥0.30 were considered the major foods associated with the dietary pattern and are marked in bold**.**

n=66,888 households (96 households were eliminated from the analysis because they did not have food consumption values)

KMO= 0.708 (interpreted as adequate)

Two dietary patterns explained 33.43% of the total variance.
